# Neurochemical Changes Following Botulinum Toxin Type A in Chronic Migraine: An LC–MS/MS and HPLC Evaluation of Plasma and Urinary Biomarkers

**DOI:** 10.3390/jcm15031208

**Published:** 2026-02-04

**Authors:** Seyma Dumur, Demet Aygun, Era Gorica, Hafize Boyaci, Bagnu Dundar, Dildar Konukoglu, Hafize Uzun

**Affiliations:** 1 Department of Medical Biochemistry, Faculty of Medicine, Istanbul Atlas University, 34203 Istanbul, Turkey; bagnu.dundar@atlas.edu.tr (B.D.); huzun59@hotmail.com (H.U.); 2 Department of Neurology, Faculty of Medicine, Istanbul Atlas University, 34203 Istanbul, Turkey; demet.aygun@atlas.edu.tr; 3 Center for Translational and Experimental Cardiology (CTEC), Department of Cardiology, University Hospital Zürich, University of Zürich, Wagistrasse 12, 8952 Schlieren, Switzerland; era.gorica@usz.ch; 4 Department of Cardiac Surgery, University Hospital Zurich, Rämistrasse 100, 8091 Zurich, Switzerland; 5 Fikret Biyal Central Biochemistry Laboratory, Istanbul University-Cerrahpasa, 34303 Istanbul, Turkey; hafize.boyaci@iuc.edu.tr; 6 Department of Medical Biochemistry, Cerrahpasa Faculty of Medicine, Istanbul University-Cerrahpasa, 34098 Istanbul, Turkey; dkonuk@yahoo.com

**Keywords:** migraine, botulinum toxin A, neurotransmitters, substance P, LC–MS/MS, HPLC

## Abstract

**Background:** Botulinum toxin type A (BoNT-A) is an established preventive therapy for chronic migraine (CM), yet the accompanying neurochemical changes remain incompletely characterized. **Objective:** To evaluate the effects of BoNT-A on plasma substance P (SP), γ-aminobutyric acid (GABA), glutamate, glutamine, and 5-hydroxytryptamine (5-HT), and on urinary 5-HT, and to explore relationships with clinical outcomes. **Methods:** In this prospective study, plasma neurotransmitters were analyzed in CM patients (n = 31) at baseline and one month after BoNT-A (155 U; PREEMPT protocol) and in healthy controls (n = 30). Plasma SP was measured using enzyme-linked immunosorbent assay (ELISA); plasma GABA, glutamate, and glutamine were quantified via liquid chromatography–tandem mass spectrometry (LC–MS/MS) with isotopically labeled internal standards; plasma and urinary 5-HT were determined by high-performance liquid chromatography (HPLC). Clinical outcomes included monthly headache frequency, Visual Analog Scale (VAS), and Migraine Disability Assessment (MIDAS). Statistical analyses applied appropriate parametric or non-parametric tests with *p* < 0.05 considered significant. **Results:** One month post-BoNT-A, headache frequency, MIDAS, and VAS were significantly reduced (all *p* < 0.001). SP levels were significantly higher after BoNT-A than at baseline and versus controls. Plasma 5-HT increased post-BoNT-A, while urinary 5-HT decreased. Plasma GABA was elevated in patients versus controls without statistical significance. Glutamine was significantly higher before treatment, whereas the Glu/Gln ratio increased after BoNT-A. Correlations revealed that higher GABA was associated with lower VAS and attack frequency post-treatment. **Conclusions:** BoNT-A provided short-term clinical improvement with distinct neurochemical changes, including increased plasma SP and 5-HT, decreased urinary 5-HT, reduced glutamine, and a higher Glu/Gln ratio. These biomarkers, particularly Glu/Gln, may serve as indicators of cortical excitability and therapeutic response in CM.

## 1. Introduction

Migraine is one of the most common and disabling conditions worldwide [1]. The pathophysiology of migraine is complex, involving the trigeminal nerve and tissues, including the meninges and blood vessels innervated by it [2]. Despite intense research over the last decade, the neurobiological basis and pathophysiology of migraine remain unclear [3,4].

Migraine treatments have evolved, and botulinum neurotoxin-A (BoNT-A) has proven to be effective in reducing headache frequency and severity in patients with migraine by reversibly inhibiting neuropeptide and neurotransmitter exocytosis from peripheral sensory neurons, thereby directly reducing peripheral sensitization [5,6]. In chronic migraine, BoNT-A is thought to exert its therapeutic effects primarily through modulation of peripheral sensitization of trigeminal nociceptors, with secondary attenuation of central sensitization, as supported by both clinical and experimental studies [7,8]. However, a significant proportion of patients either do not respond to BoNT-A and other treatments or show only a partial response [9,10,11]. Therefore, the pathophysiology of migraine needs to be elucidated to develop more effective and beneficial treatment methods.

Substance P (SP) is a neuropeptide consisting of 11 amino acids. The best-known function of SP is as a neurotransmitter and modulator of pain perception by altering cellular signaling pathways. SP is a mediator of sterile inflammation of the dura mater, thought to be the source of migraine pain [12,13].

Neurotransmitters involved in the pathogenesis of migraine include glutamate and 5-hydroxytryptamine (5-HT). A change in the balance of any of these neurological systems may lead to a higher susceptibility to migraine [14].

Gamma-aminobutyric acid (GABA) is the main inhibitory neurotransmitter. For these reasons, it has been suggested that the activation of GABA may be reduced in patients with migraine [15]. Glutamate is an excitatory neurotransmitter widely implicated in the mechanism of migraine and is thought to play a central role in central hyperexcitability and trigeminovascular activation. GABA and glutamate act like an on-off switch. They work in opposite ways. Glutamate is the main excitatory neurotransmitter in the brain [15]. GABA also works together with another neurotransmitter, 5-HT [16].

The role of glutamine in the pathophysiology of migraine has also been demonstrated by some biochemical studies. This strongly suggests that increased activity in glutamatergic systems of the brain is involved in the pathophysiology of migraine [17,18].

The literature also suggests that SP-containing neurons have intercellular and intracellular associations with GABA and 5-HT in the dorsal raphe nucleus (DRN), indicating possible interactions between GABA and 5-HT [19].

To the best of our knowledge, this is among the few studies to systematically compare correlation patterns before and after BoNT-A treatment using nonparametric correlation methods, which enhances the robustness and clinical interpretability of the findings. In this study, we aimed to investigate the effects of before and after BoNT-A administration on GABA, glutamate, glutamine, and 5-HT levels and their relationship with each other using liquid chromatography–tandem mass spectrometry (LC–MS/MS) and high-performance liquid chromatography (HPLC) methods and correlate these findings with clinical outcomes [visual analog scale (VAS) and migraine disability assessment (MIDAS)]. The techniques used in the present study are gold standard advanced analysis techniques [20].

## 2. Experimental Procedures

### 2.1. Design

This is a mono-centric, prospective, and observational clinical study.

#### 2.1.1. Sample Collection

Migraine and healthy control group samples were collected from Atlas University Hospital between 15 May 2023 and 15 December 2024. The study was approved by the Istanbul Atlas University Ethics Committee (Date: 12 May 2023, No: E-22686390-050.99-27199). Healthy controls who had no history of migraine or frequent headache and migraine patients who were diagnosed with migraine according to the International Classification of Headache Disorders (ICHD-3) criteria were included in the study [21]. The severity of headache was evaluated by the patients using the Visual Pain Scale (VAS) by giving a score between 1 and 10. Patients were given the Migraine Disability Assessment (MIDAS) score, and disability rates were determined.

#### 2.1.2. Study Groups

1. Healthy control group (n = 30);
2. Migraine patient group (n = 31) according to BoNT-A treatment:
  i  Before-BoNT-A;
  ii After-BoNT-A (One month after BoNT-A administration).

Blood samples taken in a tube with EDTA were centrifuged at 5000 rpm for 5 min, and plasma samples were separated from patients and controls and stored at −80 °C for further analysis.

For 5-HT analysis in urine, 24 h urine samples were collected from the patients before and after BoNT-A administration. During the collection phase of the study samples, preservatives (6N HCL, boric acid, etc.) were added prior to urine collection to stabilize the parameters for analysis. Urine and plasma samples to be used for the study were kept frozen at temperatures below −80 °C until the study was performed. Blood and urine sampling of patients was performed both before and after BoNT-A administration.

#### 2.1.3. Inclusion Criteria

Migraine with/without aura/chronic migraine according to the International Classification of Headache Disorders (ICHD-3) and vestibular migraine according to the Barany Association [21]; patients aged 18–60 years who had no other chronic diseases and who consented to participate in the study.

#### 2.1.4. Exclusion Criteria

Patient aged < 18 years, pregnant women, individuals who had used acute pain medication in the last 24 h, those who are constantly using migraine prophylaxis medications, invasive procedures on the skin of the head and face within 6 months, antiplatelet-anticoagulant users, acute-chronic sinusitis, patients with cranial bone defects, those with neurological or dermatological diseases, hypertensive patients, history of cardiovascular disease, hearing aid or cochlear implant users and active ear infection were excluded from the study.

Given the exploratory design and limited cohort size, a detailed stratification or exclusion based on concomitant migraine medications was not performed.

#### 2.1.5. Migraine Disability Assessment Scale (MIDAS)

It is a valid and reliable scale that retrospectively assesses migraine-related disability in different life domains covering the last 3 months [22]. The scale consists of seven questions in total. These questions are used to calculate the number of days that reduce or completely prevent work and schoolwork, household chores, and time spent with family and friends. This calculation results in the MIDAS score. The MIDAS score is calculated by summing the scores of the first five questions. Two additional questions (MIDAS A and B) measure headache frequency and average pain intensity but are not taken into account when calculating the total MIDAS score. The degree of disability of patients is categorized between I and IV. The 0–5 scores are scored as grade 1, 6–10 scores as grade 2, 11–20 scores as grade 3, and 21 and above scores as grade 4. A Turkish validity and reliability study of the scale was conducted [23].

#### 2.1.6. Visual Analog Scale (VAS)

The VAS is one of the most widely used pain measurement tools in research due to its ease of use and simple structure. This scale consists of a 100 mm vertical line. At the far-left end of the line, there is a value of 0, meaning “no pain”, while on the right side, there is a value of 10, meaning “very severe pain”. The patient is asked to mark the severity of pain on this line [24].

Migraine frequency is the number of attacks in a month.

#### 2.1.7. BoNT-A Administration

Patients were administered BoNT-A according to the Phase 3 research evaluating migraine prophylaxis therapy (PREEMPT) study protocol in the neurology clinic. In the current study, a fixed dose of 155 U BoNT-A was injected at 31 points in the frontal, temporal, occipital, and cervical regions of the head and neck [25]. Patients were asked to keep a pain diary after the procedure. Patients were evaluated in the first month. The number and severity of headaches in the last month were recorded.

### 2.2. Biochemical Analysis

#### 2.2.1. Liquid Chromatography–Tandem Mass Spectrometry (LC–MS/MS)

The LC-MS/MS Ultimate 3000 (Thermo Fisher Scientific, Waltham, MA, USA) method and recipe kit were used for quantitative analysis of plasma GABA, glutamate, and glutamine. LC-MS/MS is an analytical method that combines mass spectrometry (MS) and liquid chromatography (LC) for the quantitative and qualitative determination of analytes. The materials required for amino acid analysis in the LC-MS/MS device are mobile phase, internal standard (IS), and plasma calibrator set.

Plasma samples were injected into LC-MS/MS after preliminary preparation, and chromatograms of amino acids were obtained. The derivatized quantitative amino acid method was used as the study procedure. The analysis was performed using 5 calibrators and 2 controls of different concentrations. The procedure applied in the preparation phase of amino acid plasma and urine analysis is as follows.

A total of 70 µL plasma and 70 µL internal standard were mixed in an Eppendorf. After 15 min incubation, the supernatant was centrifuged, and the supernatant plate was taken. After evaporation under nitrogen, the derivatization reagent was added, and the supernatant was incubated again and then evaporated. A total of 100 L of solvents was added, centrifuged, and the sample was injected into LC-MS/MS. Analytes were chromatographically separated by column and electrochemically detected. The evaluation of the chromatograms was performed by the internal standard method through peak areas. In the analytical method, specific isotopically labeled internal standards for each analyte ensure robust and reliable measurement in human plasma. Mass transitions and electrospray ionization (ESI) mode of the analytes and internal standard compounds are given in Table 1.

Analyte detection was performed through compound-specific mass transitions. Analyte concentration was calculated by the internal standard method via peak areas. Calibration curves were obtained from the calibrators by plotting the “Analyte/internal standard” ratio peak area against the “Analyte” concentration. Analyte concentrations in samples and controls were calculated from the calibration curves, and results are given in µmol/L.

#### 2.2.2. High-Performance Liquid Chromatography (HPLC)

##### Analysis of 5-Hydroxytryptamine (5-HT) in Urine and Plasma

Analysis of 5-HT in urine and plasma was performed by HPLC using an UltiMate 3000 system (Thermo Fisher Scientific, Waltham, MA, USA) and a commercial kit (RECIPE Chemicals + Instruments GmbH, München, Germany).

For 5-HT analysis in urine, 24 h of urine was collected from the patients. Preservatives (6N HCL, boric acid, etc.) were added before urine collection to stabilize the parameters to be analyzed. For the 5-HT assay study of urine samples, a sample preparation procedure was performed before injection of the samples into the analytical system.

With the urine 5-HT assay procedure, sample cleanup is performed by solid-phase extraction followed by hydrolysis and pH adjustment. For this purpose, 1 mL of the urine sample and 20 µL of the Internal Standard (IS = 200 ng) were added and incubated for hydrolysis at 90–100 °C while controlling the pH. After incubation, the sample was kept at room temperature and mixed with a diluting reagent (containing a color indicator). The color of the sample was then checked, and if it was red, 1 M NaOH was added dropwise until it turned yellow. Then, the entire sample was applied to a sample preparation column for solid-phase extraction. Thus, the analytes were adsorbed on the column resin, and the resin was washed with HPLC ultrapure water and a washing solution to remove the co-adsorbed interfering substances. Finally, the analytes were separated from the resin and injected into the HPLC (20 µL). At the end of the study, the analytes were separated chromatographically by column and detected electrochemically. The evaluation of the chromatograms was performed by the IS method through peak areas. Results were given as (µg/g creatinine).

For plasma 5-HT analysis, 200 µL plasma and 10 µL internal standard were mixed, shaken, and 200 µL precipitant P was added. After this process, it was centrifuged and injected into the HPLC. Analytes were chromatographically separated by column and electrochemically detected. Evaluation of the chromatograms was performed by the internal IS method through peak areas. Results were given in µg/L.

#### 2.2.3. Enzyme-Linked Immunosorbent Assay (ELISA) Methods

In vitro quantitative determination of plasma SP concentrations was performed using a competitive ELISA (Elabscience, Cat. No. E-EL-0067, Houston, TX, USA). The sensitivity of the kit is 46.88 pg/mL, and the measurement range is 78.13–5000 pg/mL. The coefficients of intra- and inter-assay variations were 4.4% (n = 25) and 4.9% (n = 25), respectively. Analysis was performed according to the instructions of the kit manufacturers. The reaction is based on SP in the samples competing for places in the biotinylated detection antibody specific for SP and a fixed number of places in the solid-phase support. Unwanted reaction substances are washed away, and incubation procedures are applied to allow the HRP (horseradish peroxidase) solution to react. The enzyme-substrate reaction is terminated by adding the stop solution. Color change is measured spectrophotometrically at a wavelength of 450 nm. The SP concentrations of the samples were evaluated by comparing the OD of the samples with the standard curve. A total of 50 µL of patient samples, standard, and control materials were placed in the wells. The results are given as pg/mL.

##### Glutamate to Glutamine Ratio

The glutamate-to-glutamine (Glu/Gln) ratio is calculated by dividing the plasma glutamate level by the plasma glutamine level.

### 2.3. Statistical Analysis

Parametric tests were used to analyze normally distributed data, and non-parametric tests were used to analyze non-normally distributed data. The Student’s test was used to analyze quantitative data that fit the normal distribution. The Kruskal–Wallis test, which is a non-parametric test, was used for three-group comparisons that do not conform to normal distribution, and the Mann–Whitney U test was used as a post hoc analysis in the presence of significant differences between groups. The Spearman correlation test was used in the correlation analysis of the parameters analyzed between the groups. The strength of the relationship was evaluated according to the correlation coefficient (very strong correlation between 0.8 and 1.0, strong correlation between 0.6 and 0.8, moderate correlation between 0.4 and 0.6, weak correlation between 0.2 and 0.4). A two-tailed *p*-value of <0.05 was considered statistically significant.

## 3. Results

The study included 31 patients who met the criteria for chronic migraine and 30 age-sex-matched healthy volunteers. Of the patients who participated in the study, 1 (3.2%) had vestibular, 1 (3.2%) had menstrual, and 29 (93.5%) had migraine with aura type. All groups were compared in terms of demographic data, and the results are presented in Table 2. The chronic migraine patient group consisted of 8 men (25.8%) and 23 women (74.2%), and the mean age at the time of the study was 39.71 ± 10.20 years. The control group consisted of 13 healthy men (43.3%) and 17 women (56.7%) with a mean age of 44.07 ± 12.30 years. Significant decreases were found in pain frequency, MIDAS, and VAS scores at one month after BoNT-A treatment compared to before BoNT-A (*p* < 0.001, *p* < 0.001, and *p* < 0.001, respectively) (Table 3).

Study groups were also compared in terms of neuropeptide and neurotransmitter levels, and the results are presented in Table 4. SP (pg/mL) was highest in the after-BoNT-A patient group compared to both the before-BoNT-A and control groups. However, statistically significant differences were only found between the control and after-BoNT-A and before-BoNT-A and after-BoNT-A groups (0.000 and 0.016, respectively).

Plasma 5-HT (µg/L) was found to be the highest in the after-BoNT-A migraine patient group compared to both the before-BoNT-A and control groups. Plasma 5-HT levels were also higher in the before-BoNT-A group compared to the control group, but these differences were not statistically significant (*p* = 0.160). In contrast to plasma 5-HT levels, urine-5-HT (μg/g creatinine) levels were found to be the lowest in the after-BoNT-A patient group compared to both the before-BoNT-A and control groups. Urine-5-HT levels were also lower in the before-BoNT-A group compared to the control group, but these differences were not statistically significant (*p* = 0.188).

GABA (µmol/L) levels were found to be higher in the patient groups before and after BoNT-A compared to the healthy control group. However, this difference was not statistically significant (*p* = 0.095). GABA levels were also higher in the after-BoNT-A group than in the before-BoNT-A group, but these differences were also not statistically significant (*p* > 0.05).

**Figure 1:**
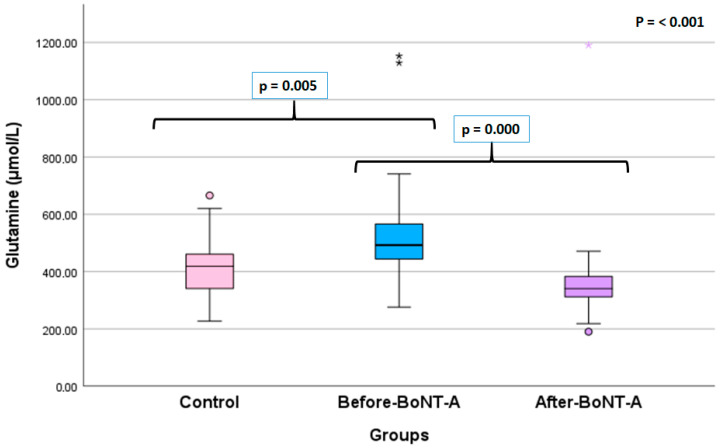
Comparison of glutamine (μmol/L) levels of the study groups (The asterisks indicate an extreme outlier).

Glutamate level was found to be the highest in the after-BoNT-A patient group and the lowest in the control group. However, this difference was not statistically significant (*p* = 0.521). On the other hand, glutamine level was significantly higher in the before-BoNT-A patient group than in both the after-BoNT-A group and the control group (*p*< 0.001 and *p* = 0.005, respectively) (Figure 1).

**Figure 2:**
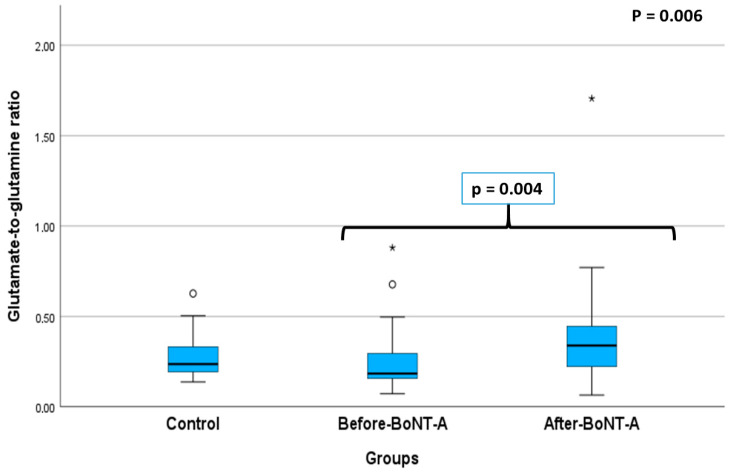
Comparison of the glutamate-to-glutamine ratio of the study groups. (The asterisk indicates an extreme outlier).

The Glu/Gln ratio was found to be lower in the before-BoNT-A patient group compared to the control group (Figure 2). However, this difference was not statistically significant (*p* = 0.300). This ratio was also significantly lower in the before-BoNT-A patient group than in the after-BoNT-A group (*p* = 0.004).

Spearman correlation analyses revealed distinct association patterns between neurotransmitter levels and clinical pain parameters across the control group and before and after BoNT-A treatment. The results of the correlation analyses were presented in Table 5. In the correlation analysis, a negative correlation was found between SP and age and between glutamine and the Glu/Gln ratio in the control group (r = −0.436, *p* = 0.016; r = −0.871, *p* < 0.001, respectively), while a positive correlation was found between glutamate and both age and the Glu/Gln ratio (r = 0.466, *p* = 0.009; r = 0.671, *p* < 0.001, respectively).

**Figure 3:**
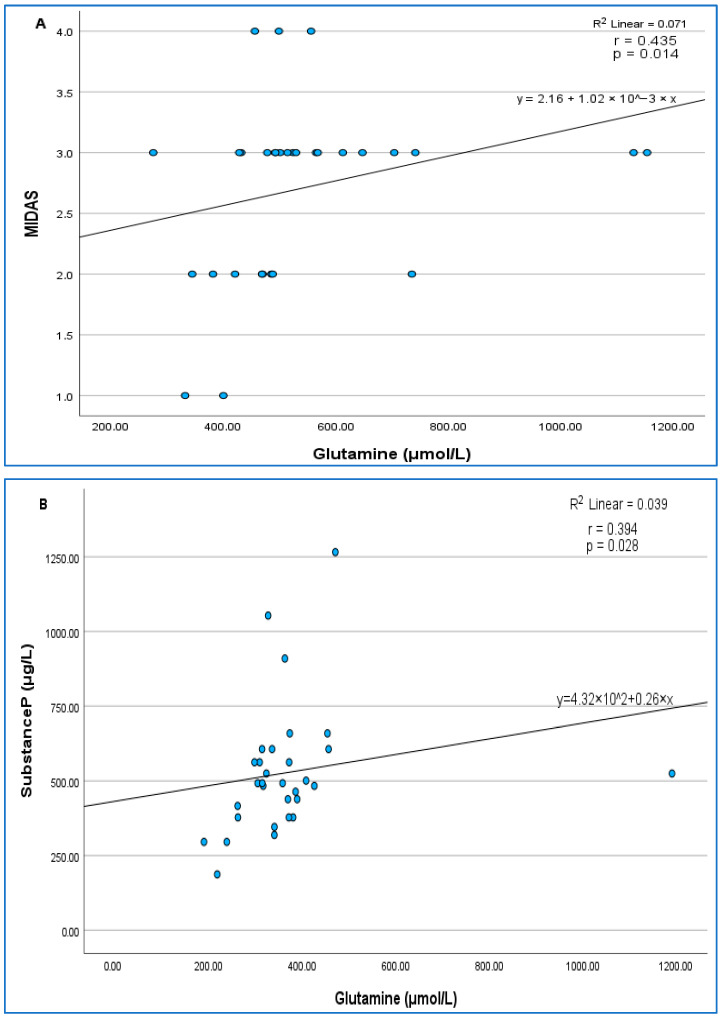
(**A**) Relationship between glutamine (μmol/L) and MIDAS score in the before-BoNT-A group. (**B**) Relationship between glutamine (μmol/L) and substance P (μg/L) levels in the after-BoNT-A group.

In the before-BoNT-A group, a negative correlation was found between 5-HT-plasma and age and frequency-monthly (r = −0.385, *p* = 0.032; r = −0.413, *p* = 0.021, respectively). A negative correlation was found between GABA and VAS (r = −0.416, *p* = 0.020). There was a negative correlation between glutamine and the Glu/Gln ratio (r = −0.637, *p* < 0.001). There was a positive correlation between glutamine and MIDAS (r = 0.435, *p* = 0.014) (Figure 3A) and a positive correlation between glutamate and the Glu/Gln ratio (r = 0.748, *p* = 0.000). A positive correlation was also found between age and frequency (r = 0.424, *p* = 0.017).

In the after-BoNT-A group, a positive correlation was found between GABA and glutamate and the Glu/Gln ratio (r = 0.414, *p* = 0.021; r = 0.374, *p* = 0.038, respectively). A positive correlation was found between SP and glutamine (r = 0. 394 *p* = 0.028) (Figure 3B). Furthermore, a negative correlation was found between plasma 5-HT and age (r = −0. 459, *p* = 0.009), and there was also a negative correlation between GABA and both VAS (r = −0.366, *p* = 0.043) and frequency-monthly (r = −0.370, *p* = 0.040).

## 4. Discussion

Current evidence on the pathogenesis of migraine remains controversial. Available treatment modalities have variable effects on migraine patients. In this study, we examined the effect of BoNT-A therapy on SP, 5-HT, glutamate, glutamine, and the Glu/Gln ratio. The main findings of this study were as follows: (i) SP was higher in the post-BoNT-A patient group compared to the pre-BoNT-A; (ii) plasma 5-HT was found to be the higher in the post-BoNT-A migraine patient group compared to the pre-BoNT-A; (iii) contrary to plasma 5-HT, urine 5-HT levels were found to be the lower in the post-BoNT-A patient group compared to both the pre-BoNT-A; (iv) glutamine level was significantly higher in pre-BoNT-A patient group than both post-BoNT-A group; and (v) Glu/Gln ratio was significantly lower in the pre-BoNT-A patient group than in the post-BoNT-A group. These findings revealed that BoNT-A treatment did not improve the clinical outcome of all migraine patients.

Patient-reported outcomes such as MIDAS and VAS are commonly used in daily practice and studies for migraine patients. In the current study, pain frequency, MIDAS, and VAS scores were found to decrease one month after BoNT-A administration. Additionally, a negative correlation was found between GABA and VAS, while a positive correlation was found between glutamine and MIDAS. Similarly, to our findings, Demiryurek et al. [26] found that VAS scores were statistically significantly lower in the first and third months after BoNT-A treatment compared to the scores obtained before treatment. Moreover, they found that the VAS scores obtained at the third month after treatment were higher than those obtained at the first month. These results show that the efficacy of BoNT-A treatment gradually decreases after the first month.

Recent research has helped researchers to better understand the pathophysiology and mechanisms underlying migraine in SP. BoNT-A alters the release of neurotransmitters (such as SP and glutamate) involved in pain transmission, reducing the number of pain signals reaching the brain and consequently preventing activation and sensitization of central neurons [27]. The effect of BoNT-A on SP is controversial [28,29,30,31]. In experimental studies, it has been reported that BoNT-A can prevent the release of SP both in vitro and in vivo [32,33,34,35,36]. It has been reported to exert these preventive effects either by preventing neurogenic plasma protein extravasation induced by sciatic nerve stimulation in rat hindlimb skin and dural plasma protein extravasation induced by different types of trigeminal pain or by preventing central SP release of peripherally injected BoNT-B. Although BoNT-A may inhibit SP transmission, Matak et al. [37] examined the role of this effect on the antinociceptive activity of BoNT-A in SP knockout mice and found that deletion of SP-encoding genes abolished BoNT-A antinociceptive activity in acute and chronic inflammatory pain as well as neuropathic pain. These observations suggest that SP signaling is involved in the antinociceptive effect of BoNT-A. In our study, serum SP levels were found to be highest in the post-BoNT-A patient group compared to both the pre-BoNT-A and control groups. There are several possible explanations for why BoNT-A is not effective one month after injection: local tissue response and irritation may cause high levels. BoNT-A injection may lead to mild tissue damage or inflammation at the injection site. This process may trigger the release of SP from nerve endings. Post-injection inflammatory responses may lead to increased levels of this neuropeptide. BoNT-A induces muscle relaxation by blocking the release of acetylcholine. However, this blockade may cause nerve endings to generate a compensatory response by increasing SP in the surrounding area. Mild pain or discomfort felt during or after injections may increase the release of SP from nerve endings. SP is released during activation of nociceptors and may increase in response to painful stimuli. In some individuals, mild inflammatory or immune responses to botulinum toxin may affect SP levels. A response by the immune system is possible, especially in repeated applications [28,38]. Similarly to our results, Heikkilä et al. [28] found no significant change in synovial fluid SP concentration in dogs treated with intra-articular BoNT A during the 8-week follow-up period. The researchers reported that the antinociceptive effect of the toxin in the joint could not be due to inhibition of SP. The conflicting effect of BoNT-A on SP may depend on the dose of treatment, the injection site, and the number of cycles. Pijpers et al. [39] conducted a randomized controlled trial in which patients with chronic migraine and medication overuse were randomly assigned to receive either botulinum toxin A (BoNT-A) or placebo. The study failed to show that BoNT-A, in addition to medication discontinuation, reduced headache days or improved patients’ quality of life compared to the placebo group.

5-HT plays a direct role in the pathophysiology of migraine, and studies on plasma and urine levels of 5-HT have shown that between migraine attacks, patients exhibit reduced plasma 5-HT levels [14]. Therefore, migraine has been thought to be a syndrome of chronic low 5-HT levels, but studies on brain 5-HT levels have yielded questionable results, although plasma levels of 5-HT do not necessarily reflect brain 5-HT levels [40]. In the literature, during the early stage of a migraine attack, blood 5-HT levels increase [40]. In line with the literature, we found that plasma 5-HT levels were higher in the before-BoNT-A group compared to the healthy control group, but these differences were not statistically significant. Interestingly, in our study, plasma 5-HT level was found to be the highest in the after-BoNT-A migraine patient group compared to both the before-BoNT-A and healthy control groups. According to the literature, the observation of low 5-HT levels in migraine has suggested a potential syndrome, but the exact results have not been clarified by investigating both brain 5-HT levels and after BoNT-A administration [41].

It has been reported that BoNT-A does not inhibit the release of GABA, an inhibitory neurotransmitter, in adult neurons [41,42]. In the current study, although plasma GABA levels were highest in the post-BoNT-A group, there was no statistically significant difference between the groups. It is possible that the increase in plasma GABA in the after-BoNT-A group is a beneficial compensatory process to alleviate migraine pain. Drinovac et al. [43,44], in different studies on rats with pain in the sciatic region, suggested that increased GABA neurotransmission mediated by GABA receptors is involved in the central antinociceptive effect of BoNT-A. In vitro experiments have shown that BoNT/A inhibits the release of GABA [45]. Our study and other studies suggest that BoNT-A may be linked to the GABAergic system in the central nervous system (CNS).

Evidence indicates that the levels of the excitatory neurotransmitter glutamate and its precursor glutamine in pain-related brain regions correlate positively with individual pain sensitivity [46]. Impaired clearance of glutamate, the major neurotransmitter released from presynaptic terminals of primary sensory afferents in the dorsal horn, including nociceptive afferents, from the perisynaptic space may play an important role during chronic pain [47]. However, to the best of our knowledge, there have been no studies examining plasma glutamine levels in migraine patients after BoNT-A. In the current study, glutamate level was found to be highest in the patient group and lowest in the control group. However, this difference was not statistically significant. On the other hand, glutamine levels were significantly higher in the before-BoNT-A patient group than in both the after-BoNT-A group and the control group, whereas glutamine levels decreased even lower than the control group after BoNT-A administration. The Glu/Gln ratio was found to be lower in the patient group before BoNT-A compared to the control group. However, this difference was not statistically significant. As expected, this ratio was significantly lower after BoNT-A administration. In a larger cohort, Zielmann et al. [48] reported that glutamate levels were increased in the occipital cortex of patients with migraine. The Glu/Gln ratio is elevated in both the occipital cortex and the right thalamus of migraine patients [49]. Noseda et al. [50] were able to demonstrate a direct effect of the Glu/Gln ratio on the activity of thalamic trigeminovascular neurons. Steel et al. [51] have found a positive correlation between GABA and the Glu/Gln ratio in the resting human brain. The correlation between GABA and the Glu/Gln ratio in the human brain suggests that the concentrations of excitatory and inhibitory neurotransmitters are balanced in the human brain. In our study, in the after-BoNT-A group, a positive correlation was found between GABA with glutamate and the Glu/Gln ratio. The close interactions between GABA, glutamate, and the Glu/Gln ratio after BoNT-A administration suggest that these neurotransmitters work together in complex ways in BoNT-A modulation, not alone. Many of the proposed mechanisms underlying the analgesic effects of the toxin are based on in vivo animal models and in vitro culture systems showing that BoNT-A can suppress the local release of substances involved in pain and vasodilation, including glutamate and GABA. Importantly, concomitant medication use, including acute migraine treatments such as triptans, may influence circulating neurotransmitter and neuropeptide levels and therefore represents a potential confounding factor. The absence of systematic stratification based on medication use constitutes a limitation of this pilot study and should be addressed in future, adequately powered investigations.

Although it presents limitations due to a small sample size and a short follow-up period, its strength lies in the comprehensive assessment of multiple neurotransmitters and patient-reported outcomes, providing novel insights into the effects of BoNT-A in migraine.

## 5. Conclusions

This study represents a preliminary, hypothesis-generating pilot investigation of urinary and plasma biomarkers following botulinum toxin type A (BoNT-A) treatment in migraine patients. Owing to the exploratory design and limited sample size, the clinical and biological relevance of the observed biomarker changes cannot be established, and the findings should be interpreted with caution.

Clinical responses to BoNT-A were heterogeneous, with no uniform improvement observed across the study population, underscoring inter-individual variability in treatment response. The detected alterations in plasma GABA, 5-HT, substance P, glutamate, and glutamine levels, as well as urinary 5-HT, identify candidate molecules that may merit further investigation in the context of migraine pathophysiology. The glutamate/glutamine ratio emerged as a potential signal in this cohort; however, its utility as a biomarker requires confirmation in larger, well-powered studies.

Overall, these results primarily serve to inform the design of future studies aimed at validating biomarkers and clarifying the mechanisms, efficacy, and predictors of response associated with BoNT-A therapy.

## Figures and Tables

**Table 1:** Mass transitions and electrospray ionization (ESI)-mode of the analytes and internal standard compounds.

Analyte/IS	ESI-Mode	Quantifier MRM		Qualifier MRM	
		**Precursor** **[*m*/*z*]**	**Product** **[*m*/*z*]**	**Precursor** **[*m*/*z*]**	**Product** **[*m*/*z*]**
Glutamate	Positive	148	84	148	102
Glutamine	Positive	147	84	147	130
GABA	Positive	104	87	104	87

**Table 2:** Demographic characteristics of chronic migraine patients and healthy control groups.

	Control (n = 30)	Chronic Migraine (n = 31)	*p*-Value
Gender (F/M)	17/13	24/7	0.241
Age (Year)	44.07 ± 12.301	39.71 ± 10.202	0.068

**Table 3:** Pain frequency (monthly), Migraine Disability Assessment (MIDAS) grade, and Visual Pain Scale (VAS) scores of migraine patients before and after BoNT-A.

	Before-BoNT-A	After-BoNT-A	*p*-Value
Pain frequency, monthly	12.52 ± 6.971	1.32 ± 1.275	<0.001
MIDAS	2.71 ± 0.739	0.74 ± 0.815	<0.001
VAS	8.23 ± 1.309	1.97 ± 1.722	<0.001

**Table 4:** Comparison of laboratory results of the chronic migraine patients and control groups.

	Control (n = 30)	Before BoNT-A (n = 31)	After BoNT-A (n = 31)	*p*-Value
Substance P (pg/mL)	314.30 (234.37–401.03)	348.76 (275.42–524.81) ^c,^*	492.29 (377.70–606.55) ^a,^***	<0.001
5-HT plasma (µg/L)	108 (85.15–151.22)	118.60 (62.80–209.60)	152.60 (67.70–264.20)	0.160
5-HT urine (ug/g creatinine)	127 (88.92–151.25)	103 (66.90–127.00)	100 (73.90–133.00)	0.188
GABA (µmol/L)	0.15 (0.13–0.17)	0.18 (0.13–0.24)	0.19 (0.14–0.24)	0.095
Glutamate (µmol/L)	98.75 (90.54–114.62)	104.36 (81.08–141.95)	117.02 (84.81–144.31)	0.521
Glutamine (µmol/L)	418.59 (339.86–462.98) ^b,^**	492.56 (431.80–567.68) ^c,^***	340.68 (309.05–385.90)	<0.001
Glu/Gln ratio	0.235 (0.191–0.337)	0.183 (0.152–0.302) ^c,^**	0.338 (0.217–0.446)	0.006

5-HT: 5-hydroxytryptamine, GABA: Gamma amino butyric acid. ^a^ control vs. after BoNT-A; ^b^ control vs. before BoNT-A; ^c^ before BoNT-A vs. after BoNT-A. * *p* < 0.05, ** *p* < 0.01, *** *p* < 0.001.

**Table 5:** Correlation analyses of neurotransmitters and clinical variables.

**(A) Control Group**										
**A**		**Substance P**		**Glutamate**	**Glutamine**	**Age**	**Glutamate/Glutamine Ratio**			
Substance P		—								
Glutamate		−0.004		—						
Glutamine		0.066		−0.358	—					
Age (years)		−0.436 *		0.466 **	−0.138	—				
Glutamate/Glutamine ratio		−0.071		0.671 **	−0.871 **		—			
**(B) Before BoNT-A**										
**B**		**5-HT (Plasma)**	**GABA**	**Glutamate**	**Glutamine**	**Age**	**Sex**	**MIDAS**		
5-HT (Plasma)		—								
GABA		0.220	—							
Glutamate		0.262	0.335	—						
Glutamine		−0.247	0.233	−0.096	—					
Age (years)		−0.385 *	0.085	−0.111		—				
Sex		0.016	0.095	−0.256			—			
MIDAS score		−0.195	−0.348	−0.126	0.435 *	−0.285	0.028	—		
VAS score		0.038	−0.416 *	0.144	0.041	−0.278	−0.286	0.406 *		
Pain frequency (monthly)		−0.413 *	−0.334	−0.231	0.183	0.424 *	−0.004	0.096		
Glutamate/Glutamine ratio		0.272	−0.008	0.748 **	−0.637 **	−0.228	−0.132	−0.233		
**(C) After BoNT-A**										
**C**	**Substance P**	**5-HT (Plasma)**	**GABA**	**Glutamate**	**Glutamine**	**Age**	**Sex**	**MIDAS**	**VAS**	**Pain Frequency (Monthly)**
Substance P	—									
5-HT (Plasma)	0.033	—								
GABA	0.070	−0.314	—							
Glutamate	0.029	−0.009	0.414 *	—						
Glutamine	0.394 *	−0.314	−0.027		—					
Age (years)	−0.060	−0.459 **	0.158			—				
Sex	−0.157	−0.016	−0.058				—			
MIDAS score	0.024	0.110	−0.272					—		
VAS score	−0.169	0.009	−0.366 *	−0.097	0.064			0.862 **	—	
Pain frequency (monthly)	−0.155	0.008	−0.370 *	−0.228	0.102			0.809 **	0.906 **	—

Data are presented as correlation coefficients (r). * *p* < 0.05, ** *p* < 0.01.

## Data Availability

The raw data can be obtained on request from the corresponding author.
